# Developmental Exposure of Mice to Dioxin Promotes Transgenerational Testicular Inflammation and an Increased Risk of Preterm Birth in Unexposed Mating Partners

**DOI:** 10.1371/journal.pone.0105084

**Published:** 2014-08-15

**Authors:** Kaylon L. Bruner-Tran, Tianbing Ding, Kallie B. Yeoman, Anthony Archibong, Joe A. Arosh, Kevin G. Osteen

**Affiliations:** 1 Women's Reproductive Health Research Center, Department of Obstetrics and Gynecology, Vanderbilt University School of Medicine, Nashville, Tennessee, United States of America; 2 Department of Pathology, Microbiology and Immunology, Vanderbilt University School of Medicine, Nashville, Tennessee, United States of America; 3 Department of Physiology, Meharry Medical College, Nashville, Tennessee, United States of America; 4 Department of Veterinary Integrative Biosciences, Texas A & M University College of Veterinary Medicine, College Station, Texas, United States of America; The University of Manchester, United Kingdom

## Abstract

TCDD (2,3,7,8-tetrachlorodibenzo-p-dioxin, commonly known as dioxin) is a ubiquitous environmental contaminant and known endocrine disruptor. Using a mouse model, we previously found that adult female mice exposed *in utero* to TCDD (F1 generation) as well as multiple subsequent generations (F2-F4) exhibited reduced fertility and an increased incidence of spontaneous preterm birth. Additional studies revealed that male F1 mice with a similar in utero/developmental TCDD exposure also exhibited diminished fertility and conferred an increased risk of preterm birth to their unexposed mating partners. Herein, we extend these previous observations, reporting that reduced fertility in male F1 mice is linked to testicular inflammation which coincides with apoptosis of developing spermatocytes, sub-fertility and an increased risk of preterm birth in their unexposed mating partners. Significantly, in the absence of additional toxicant exposure, testicular inflammation and reduced fertility persisted in F2 and F3 males and their control mating partners also frequently exhibited spontaneous preterm birth. Although a steady, global decline in male fertility has been noted over the last few decades, the reasons for these changes have not been firmly established. Likewise, the PTB rate in the U.S. and other countries has paralleled industrial development, suggesting a possible relationship between environmental toxicant exposure and adverse pregnancy outcomes. Most current clinical strategies to prevent preterm birth are focused solely on the mother and have yielded limited benefits. In contrast, our studies strongly suggest that the preconception testicular health of the father is a critical determinant of pregnancy outcomes in mice. Future clinical studies should examine the potential contribution of the male to gestation length in women and whether efforts to reduce the incidence of preterm birth should be initiated in both parents prior to pregnancy.

## Introduction

Exposure to environmental toxicants and pharmaceutical chemicals is common across the human lifespan; thus, understanding the potentially negative impact of exposure to bioactive chemicals is paramount to protecting our reproductive health [1]. Of particular concern, recent animal models have shown that developmental exposure of a single generation to endocrine disrupting environmental toxicants can negatively impact reproductive capacity trangenerationally, likely due to epigenetic inheritance (reviewed by [2]). The tragic history of exposure to the pharmacologic agent diethylstilbestrol (DES) provides clear evidence that developmental exposure to an endocrine disrupting chemical can have multi-generational effects on human health (reviewed by [3]). Given this background, the rapidly emerging concept that the environmental exposure history of paternal and maternal ancestors may negatively affect an individual's current reproductive health demands a shift in our medical assessments and treatments of infertility. More specifically, since ancestral toxicant exposures cannot be changed, it is imperative that we begin to identify core reproductive processes that are negatively impacted by familial toxicant exposure such that targeted therapies to preserve male and female fertility and avoid adverse pregnancy outcomes can be designed.

More than 80,000 chemicals have been released into our environment since the Toxic Substance Control Act (TSCA) of 1976, however; only a limited number of these potentially harmful compounds have been investigated under controlled experimental conditions [4], [5]. In the absence of sufficient safety information, the recognition that natural and manufactured chemicals are capable of disrupting reproductive success has recently prompted the American Society of Reproductive Medicine and the American College of Obstetrics and Gynecology to release guidelines designed to raise awareness of environmental toxicants among clinical caregivers of reproductive age women [6]. Although the safety profile of many chemicals remains to be determined, 2,3,7,8-tetrachlorodibenzo-p-dioxin (TCDD) is known to impair male and female fertility due to the ability of this toxicant to not only disrupt endocrine signaling [7] but also modulate critical aspects of immune cell function [8] [9]. The principal mechanism of action of TCDD is related to the binding of this toxicant to the aryl hydrocarbon receptor (AhR) [8], an orphan nuclear receptor which is expressed in the reproductive tract of both humans and rodents [10]–[12]. In addition to TCDD, other structurally related toxicants also bind the AhR, including polychorinated dibenzodioxins (PCDDs), polychlorinated dibenzofurans (PCDFs) and co-planar (non—ortho-substituted) polychlorinated biphenyls (PCBs; [13]–[15]). Importantly, whereas AhR-binding toxicants frequently act as disruptors of reproductive function, male and female AhR knockout mice also exhibit altered reproductive tract development and reduced adult fertility [16], [17], implicating endogenous ligands for this receptor as necessary for normal reproduction.

Utilizing TCDD as a prototypical AhR agonist, our laboratory demonstrated that a single exposure of pregnant mice to TCDD reduced the fertility of female offspring for multiple generations whereas identically exposed animals able to achieve pregnancy as adults exhibited an elevated risk of spontaneous preterm birth (PTB) [18]. Suggesting that both endocrine and immune disruption had occurred, female offspring with a direct (F1-F2) or indirect (F3) TCDD exposure exhibited a doubling of the incidence of spontaneous PTB in the presence of either a viral infection or following a low-dose challenge with lipopolysaccharide (LPS). Significantly, exposure of control mice to the same viral or bacterial challenge did not lead to pregnancy loss, demonstrating that female mice with a history of toxicant exposure have an increased sensitivity or reactivity to a second inflammatory challenge [18]. In follow-up studies, we examined the fertility of male mice with a history of TCDD exposure as well as their contribution to successful pregnancy outcomes. In agreement with data from other groups (reviewed by [19]), we found that developmental toxicant exposure of male mice (F1) negatively impacted adult fertility. However, it was somewhat unexpected that mating fertile F1 males to control female mice resulted in a similar rate of PTB as observed with toxicant exposed females mated to control males [20]. Significantly, multiple studies have demonstrated that paternally expressed genes predominate within the placenta [21]–[23]. Additionally, recent evidence suggests that the timing of parturition is regulated by placental inflammatory signals [24]. Therefore, we next examined the influence of a preconception anti-inflammatory diet on pregnancy outcomes. Providing a fish oil supplemented diet to F1 males prior to conception improved male fertility, placental function and normalized gestation length in their female mating partners [25]. Taken together, our prior studies suggested that TCDD-mediated disruption of preconception testicular health may be biologically linked to the altered placental function that we associated with the paternal-derived risk of PTB. Therefore, the primary goal of the current study was to examine the effect(s) of early life TCDD exposure on testicular health, sperm quality and the reproductive performance of male mice (F1 generation). Equally significant, since TCDD is a known epigenetic modifier [18], [26], we also examined the F3 generation, the first without a direct toxicant exposure, for the same reproductive endpoints. Clearly, the ability of a toxicant to induce inheritable changes to the epigenome is dependent upon exposure during a brief developmental window that is sensitive to reprogramming. The Skinner laboratory has demonstrated a transgenerational, negative effect of TCDD on adult reproductive function following exposure during the time of sex determination in rats [26], [27]. Our results indicate that a single, acute TCDD exposure given during pregnancy after sex determination is also capable of altering reproductive performance in adult offspring (F1 males). Furthermore, these same parameters were adversely affected in the indirectly exposed F3 males, which is indicative of transgenerational inheritance of the phenotype. Finally, we demonstrate that biomarkers of inflammation within the testis of toxicant exposed mice are a signature of ancestral toxicant exposure and appear to be predictive of a risk for adverse pregnancy outcomes in unexposed female mating partners.

## Materials and Methods

### Animals

Young adult (8–10 weeks) male and female C57bl/6 mice were purchased from Harlan Spraque-Dawley Laboratories (Indianapolis, IN). Animals were housed in Vanderbilt University's Barrier Animal Care Facility (free of mouse pathogens including MPV and MNV) according to National Institutes of Health and institutional guidelines for laboratory animals. All animals received low phytoestrogen rodent chow (Picolab 5VO2, Purina TestDiets, Richmond, IN) and water ad libitum. Animal rooms were maintained at a temperature of 22–24°C and a relative humidity of 40–50% on a 12-hour light:dark schedule. Animals were acclimated at least one week prior to initiation of studies. Experiments described herein were approved by Vanderbilt University's Institutional Animal Care and Use Committee in accordance with the Animal Welfare Act.

### Chemicals

TCDD (99%) in nonane solution was obtained from Cambridge Isotope Laboratories (Andover, MA). All other chemicals were obtained from Sigma-Aldrich (St. Louis, MO).

### In utero TCDD Exposure

Virgin C57bl/6 females (N = 20), aged 10–12 weeks, were mated with intact males of similar age. Upon observation of a vaginal plug, females were separated and denoted as day 0.5 of pregnancy (E0.5). Pregnant mice (F0) were exposed to TCDD (10 µg/kg) in corn oil or vehicle alone by gavage at 1100 hours CST on E15.5 (when organogenesis is complete). This in utero plus lactational exposure paradigm results in direct exposure of the feti (F1 mice) as well as direct exposure of the fetal germ cells, which have the potential to become the F2 generation. This dose of TCDD reflects the more rapid clearance of this toxicant in mice compared to humans and is well below the LD50 for adult mice of this strain (230 µg/kg) [28]. TCDD given at this time and dose is not overtly teratogenic and gestation length was not affected in the F0 animals; pups (F1 mice) were typically born on E20.

### Monitoring Pregnancy

A single control female was placed with a single male (with or without a direct/ancestral TCDD exposure) and monitored for the presence of a vaginal plug (E0.5) each morning. Following the identification of a plug, the male was removed. Females were weighed prior to mating and again on E16.5, when they were examined for signs of pregnancy (weight gain, nipple prominence). Beginning at this time, pregnant females were monitored daily until delivery. Male partners of females in which a plug was observed, but which did not demonstrate signs of pregnancy were mated to a different female. Males which produced 3 positive vaginal plugs, but no pregnancy were considered infertile.

Parturition in C57bl/6 mice normally occurs 19.5 days after identification of a vaginal plug [29]; therefore, term parturition is considered E20. For our studies, pups are considered preterm if born prior to E19 [20], [25], a more stringent definition than that used by other groups (ie [30], [31]).

### Euthanasia of Males, Collection of Testes and Analysis of Sperm Concentration

Following a minimum of 72 hrs after mating, males were euthanized by cervical dislocation under isoflurane anesthesia. Testes were excised, weighed and epididymal caudal sperm was collected and quantified by standard methodology [32]. Briefly, caudal sperm were collected into 2 mL prewarmed PBS. Cells were diluted 1∶20 and 10 uL of the dilution counted using a hemocytometer. Two counts were taken for each sample and the average used to determine sperm concentration for each animal using the following equation: Sperm number = (mean count×dilution factor)/cauda weight (mg). After removal of the epididymis, the remainder of one testis was formalin-fixed and subjected to paraffin-embedding and sectioning (5 um). The remainder of the second testis was processed for prostaglandin E_2_ (PGE_2_) analysis as described below.

### Assessment of Sperm Morphology

**Figure 1:**
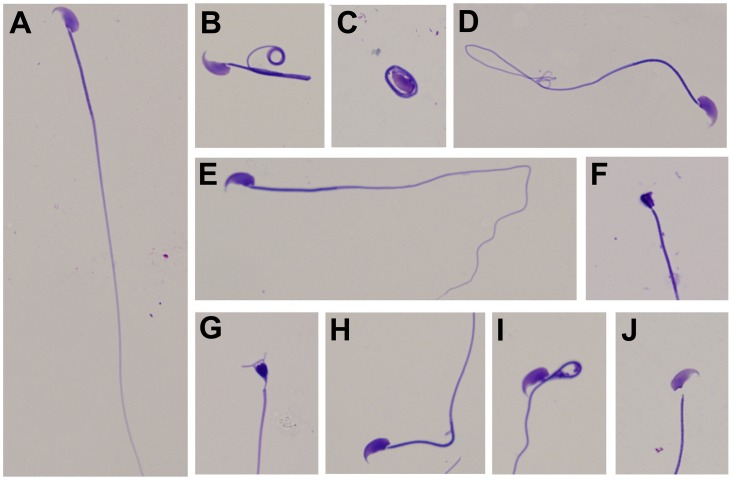
Light microscopy of normal and abnormal murine spermatozoa. All spermatozoa were classified by standard morphologic assessment. Normal spermatozoan (A); common tail defects (B–E); acrosome defect (F); misshapen head (G); mid-piece defects (H, I) and decapitated sperm (J). Magnification, 1000x.

Sperm smears were methanol fixed and stained using Stat III Andrology Stain (Mid-Atlantic Diagnostics, Mt. Laurel, NJ) which meets the Kruger Strict Criteria for analysis of Sperm Morphology. Sperm morphologies were assessed by standard methods [33], [34] by three individuals (KBT, AA and KBY) blinded to the treatment groups. Briefly, smears were prepared on histological slides and allowed to dry. After staining, 200 spermatozoa/mouse were analyzed under oil immersion (1000× magnification) using an Olympus BX51 microscope system. Morphological abnormalities were classified by careful examination of the head, tail, acrosome and mid-piece (**Figure 1**).

### Lipopolysaccharide Exposure

LPS (1 mg/mL; Enzo Life Sciences, E. coli serotype 055:B5 S-form, TLR grade) was diluted in sterile PBS immediately prior to intraperitoneal injection (25 g needle) at a dose of 200 ug/kg.

### Immunohistochemistry

Immunohistochemical localization of selected proteins was conducted in our laboratory using commercially available antibodies and the Vectastain Elite ABC kit (Vector Laboratories Inc, Burlingame, CA) according to the manufacturer's protocol. Antibodies and concentrations used were: prostaglandin dehydrogenase (PGDH; Cayman Chemicals #160615; used at 1∶500); F4/80 (a macrophage specific marker; eBioscience #14-4801-85; used at 1∶400); cleaved Caspase-3 (a marker of apoptosis; Cell Signaling Technologies #9661; used at 1∶100) and Aryl Hydrocarbon Receptor (AhR; Santa Cruz sc-8089; used at 1∶500). Commercially available, species-specific biotinylated secondary antibodies were used per the manufacturer's recommendation. All slides were viewed using an Olympus BX51 microscope system and images captured using an Olympus DP71 digital camera.

### TUNEL Staining

In addition to immunostaining for cleaved caspase-3, apoptosis was evaluated by detection of DNA fragmentation using TUNEL assay as previously described [35]. Briefly, a commercially available kit (DeadEnd Colorimetric TUNEL System; Promega) was used according to manufacturer's instructions. The assay involves identification of nicks in the DNA, which are identified by terminal deoxynucleotidyl transferase.

### Testicular PGE_2_ Extraction and Analysis by ELISA

Intracellular PGs from whole testis were extracted as described by others [36], [37]. Fresh whole testis samples were homogenized in ice-cold Tris-buffer (pH 7.4) containing indomethacin (100 µM) using a polytron homogenizer on ice. The homogenate was centrifuged at 50,000×g for 1 h at 4°C using a floor model Sorvall Discovery M150 ultracentrifuge. Supernatants were collected and the PGE_2_ concentration analyzed using a commercially available ELISA kit (Cayman Chemicals) according to the manufacturer's instructions. Intra- and interassay coefficients of variation (CVs) were determined at multiple time points on the standard curve as described by the manufacturer in each assay and compared between assays. For the PGE_2_ assay, the sensitivity or minimal detection limit was 7.8 pg/ml; intra-assay CV was 4.2% and interassay CV 12.4%.

### Radioimmunoassay of Free Testosterone

Serum samples were analyzed for testosterone concentrations by RIA in the Center for Research in Reproduction Ligand Assay and Analysis Core at the University of Virginia. The sensitivity of testosterone assay was 5 ng/dL and the intra- and inter-assay coefficients of variation were 4.4 and 6.4%, respectively.

### Computer-Assisted Assessment of AhR Staining Intensity

AhR intensity was semi-quantitatively measured using MetaMorph Microscopy Automation & Image Analysis Software. Briefly, the maximum color threshold for AhR staining was determined across all slides, then individual sperm were analyzed and the integrated intensity of the region was configured and measured.

### Assessment of Leukocyte Infiltrates

After staining with F4/80 as described above, macrophage infiltrates within the testes were enumerated by two individuals (KBT, KBY) in five high power fields (HPF) per group (400× magnification) using a method similar to that described by others for assessment of immune cell populations in tumors [38]. Fields were selected after low power scanning to identify the most highly stained areas on each slide. The same 5 fields from each group were enumerated by both observers and the resulting 10 numbers were averaged in order to semi-quantitatively determine the number of macrophages present within control and F1/F3 mice.

### Semi-quantitative Assessment of TUNEL and PDGH staining

For calculation of H-score, a minimum of 5 slides per sample were selected at random and 2 fields from each slide photographed at ×200 magnification. Staining intensity within spermatocytes was independently scored as 0, 1, 2 or 3, corresponding to the presence of negative, weak, intermediate or intense staining, respectively. The same fields from each group were enumerated by two observers and the average of percent positive cells at each level of intensity was determined, and the H-score calculated based on the formula: H-score = (% of cells stained at level 1 intensity × 1)+(% of cells stained at level 2 intensity × 2) + (% of cells stained at level 3 intensity × 3). In this manner, an H-score of between 0 and 300 is obtained where a score of 300 is equivalent to 100% of cells exhibiting maximum staining.

### Statistical Analysis

Analyses were performed with GraphPad Prism©5 software and presented as mean±SEM. The statistical difference between samples was determined using one-way analysis of variance (ANOVA) followed by Tukey's post-hoc test. P<0.05 were considered significant.

## Results

### TCDD Exposure is Associated with Reduced Fertility and Preterm Birth

We previously reported that adult male mice with a history of developmental TCDD exposure exhibited reduced fertility and conferred an increased risk of delivering preterm to their control mating partners [20]. Extending these earlier observations, herein we demonstrate that male mice with either a direct (F1–F2) or indirect (F3 via the paternal germline) TCDD exposure exhibit a significant reduction in both fertility (p≤0.001) and gestation length (p≤0.0001) compared to control mice (**Table 1**). Specifically, approximately 50% of all F1–F3 males were unable to impregnate their mating partner while those partners that became pregnant frequently (33–38%) experienced spontaneous delivery prior to E19.0 (**Table 1**). In contrast to mice with a toxicant exposure history, infertility was rare in control mating pairs and PTB was never observed among these animals (**Table 1**). Similarly, descendants of control mating pairs did not exhibit either infertility or PTB (data not shown).

**Table 1:** Impact of Direct (F1-F2) or Indirect (F3) Paternal TCDD Exposure on Pregnancy.

MALE EXPOSURE	PREGNANCY RATE*		LENGTH	OF	GESTATION	(days)		
			PT PT	FT	FT FT	FT	Avg	
		*p* **	***17.5 18.5***	***19.0***	***19.5 20.0***	***20.5***		*p* **
**Control**	38/40 (95%)				5% 90%	5%	20	
								
**TCDD**								
**F1**	29/62 (47%)	<0.001	14% 24%	62%			18.6	<0.0001
**F2**	12/25 (48%)	<0.001	33%	67%			18.8	<0.0001
**F3**	14/28 (50%)	<0.001	7% 28%	28%	28% 7%		19.0	<0.0001

*Mice achieving 3 vaginal plugs, but no pregnancy, were considered infertile.

**compared to control mating pairs.

PT = preterm; FT = Full-term.

### Assessment of Sperm Number and Morphology

**Figure 2:**
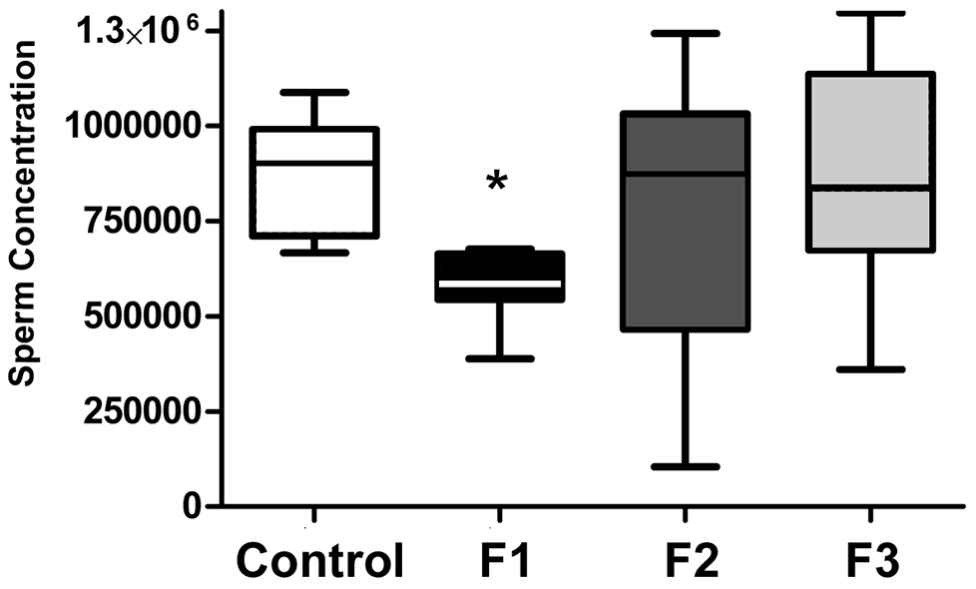
Analysis of sperm concentration in control and toxicant-exposed mice. Box plot of sperm number/mg caudal weight. Center lines indicate the median number. Columns and vertical bars indicate the 25–75 percentiles and 10–90 percentiles, respectively. Data from multiple animals per group is presented (Control, N = 7; F1, N = 6; F2, N = 8; F3, N = 13). One additional F1 male was azoospermic and was excluded from the data presented. *p = 0.001.

**Figure 3:**
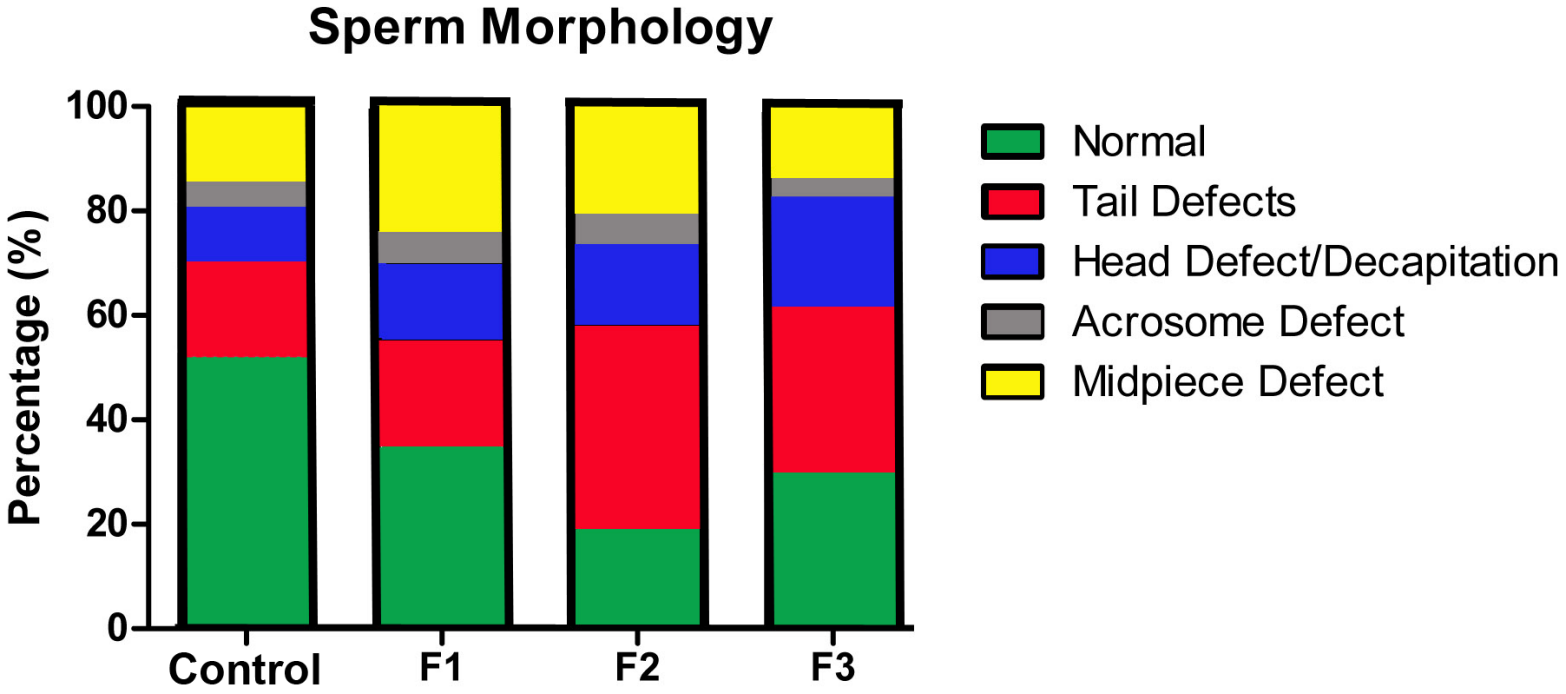
Analysis of sperm morphology of control C57bl/6 mice, F1 males and two generations of descendants. Unexposed control mice exhibited the highest number of morphologically normal sperm (53%). There was a significant reduction in the number of morphologically normal sperm in males with a direct toxicant-exposure history (F1: 36%; *p* = 0.01 and F2: 20%; *p* = 0.0001, compared to control). Mice with only an indirect exposure also exhibited significant reductions in morphologically normal sperm (F3: 31%; *p* = 0.0001, compared to control). Tail defects were the most common abnormality in all groups, while spermatocytes from mice with a history of TCDD exposure (direct or indirect) exhibited a slight increase in head, mid-piece and acrosome defects compared to control mice. A minimum of 200 cells were analyzed per animal. N≥6 for all groups.

Based on the transgenerational observations of both infertility and PTB described above, we next determined whether reduced sperm quantity and/or quality was a factor in the infertility rate of toxicant-exposed males and/or the poor pregnancy outcomes in their unexposed mating partners. The overall success rate for control mating pairs was 95% (**Table 1**) and control males in our study exhibited normal sperm numbers (**Figure 2**) with the percent of morphologically normal sperm within the range reported by others for adult C57bl/6 mice [39], [40] (**Figure 3**). Regardless of exposure (or lack of exposure), tail defects were the most common morphological abnormality. In contrast to control males, the pregnancy success rate and sperm number were significantly reduced in F1 males (**Table 1** **and** **Figure 2**). Interestingly, the *average* sperm number for F2 and F3 males was similar to control males; however, there was considerable variation in the sperm concentration in individual mice within these groups (**Figure 2**). Although the percent of morphologically normal sperm was lower in F2 and F3 males compared to F1 males, the fertility rate was similar across all exposure groups (**Table 1** **;** **Figure 3**). The average gestation length of pregnant control females mated to F1-F3 males increased incrementally with each successive paternal generation; however this improvement did not reach significance (**Table 1**).

### AhR Expression in Spermatocytes

**Figure 4:**
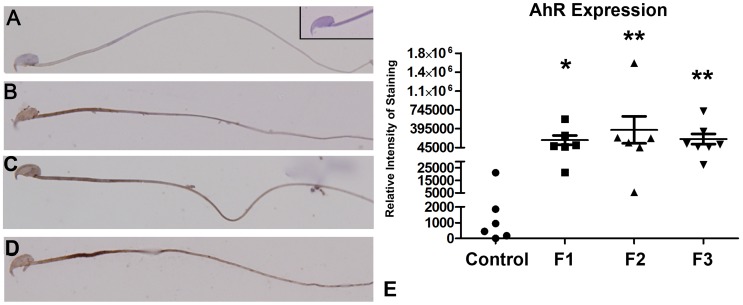
Immunolocalization of AhR protein in caudal sperm smears from adult control, F1, F2 and F3 males. AhR protein expression was minimal in control animals (A), but this protein was highly expressed in F1 (B) F2 (C) and F3 (D) sperm. Computer assisted AhR intensity (E) was determined using a minimum of 10 individual sperm from each of 6 animals per group. Original magnification, 1000x. **Inset**: Primary antibody omitted using sperm from a control male. Compared to control, *p = 0.001; **p = 0.0006.

Although the AhR is expressed throughout the male reproductive tract and appears necessary for normal sperm development, spermatocytes themselves normally express only low levels of this protein [40]. Relevant to the current study, increased spermatocyte AhR expression has been linked to infertility in humans [10] and toxicant exposure in adult rats [40]. We therefore subjected caudal sperm smears to immunolocalization of the AhR. In agreement with data described by Hansen et al [40], sperm from control animals exhibited minimal AhR expression, which was localized to the acrosome and mid-piece (**Figure 4A**). However, sperm obtained from mice with a direct (F1-F2) or indirect (F3) TCDD exposure history had significantly (p≤0.001) increased immunolocalization of AhR compared to control cells (**Figure 4B–D**).

### TCDD Exposure is Associated with a Hyper-Inflammatory Testicular Phenotype

**Figure 5:**
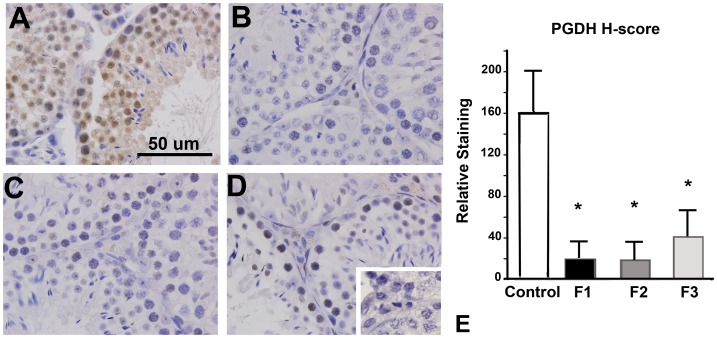
Immunolocalization of PGDH in adult testis of control, F1, F2 and F3 males. PGDH was abundant in testis from control males (A), but markedly reduced in F1 (B) and F2 (C) males. PGDH immunolocalization remained low in the testis of F3 males (D). Semi-quantitative analysis of PGDH staining is shown in Panel E. Compared to control samples, *p*<0.001 for all toxicant exposed mice. Results are representative of at least 6 animals per group and from ≥3 more litters/group. Original magnification, 400x. **Inset**: Primary antibody omitted using the same tissue shown in Panel D.

**Figure 6:**
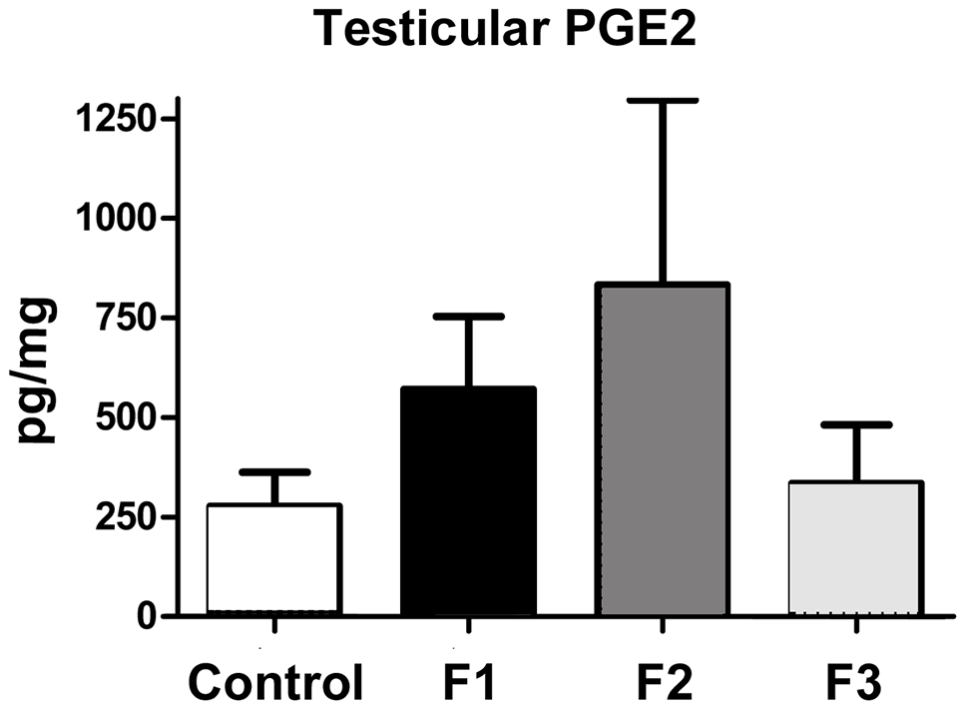
ELISA of PGE_2_ in whole testis of adult control, F1, F2 and F3 males. Only low levels of PGE_2_ were detected in the testis of all control animals. In mice with a toxicant exposure history, there was a clear trend toward increased expression of PGE_2_; however, production of this prostaglandin was highly variable and did not reach significance (compared to control: F1, *p* = 0.07; F2, *p* = 0.10; F3, *p* = 0.16). N = 6 for all groups.

We have previously demonstrated a TCDD-associated loss of prostaglandin dehydrogenase (PGDH) within the placenta arising from F1 males [25]. Since PGDH is the enzyme which catabolizes PGE_2_ to its inactive form, reduced expression of this enzyme is associated with increased local inflammation [41]. Therefore, we next examined the expression of PGDH and PGE_2_ within the testis of control and toxicant exposed male mice. Immunohistochemical localization of PGDH expression revealed a near absence of this protein in testis obtained from F1 and F2 males compared to unexposed animals (**Figure 5A–C**). Testes from F3 males exhibited only minimal immunolocalization of PGDH (**Figure 5D**). Quantitative assessment of PGE_2_ in whole testicular samples of testis of control animals revealed a low level of this enzyme in all control animals while testes from mice with a history of TCDD exposure exhibited variable PGE_2_ levels (**Figure 6**). Although changes in PGE_2_ levels among toxicant exposed mice did not reach statistical significance, a clear trend of increased PGE_2_ production was observed and correlated with reduced expression of PGDH. Additionally, among individual animals, mice with the lowest sperm number exhibited the highest level of testicular PGE_2_.

**Figure 7:**
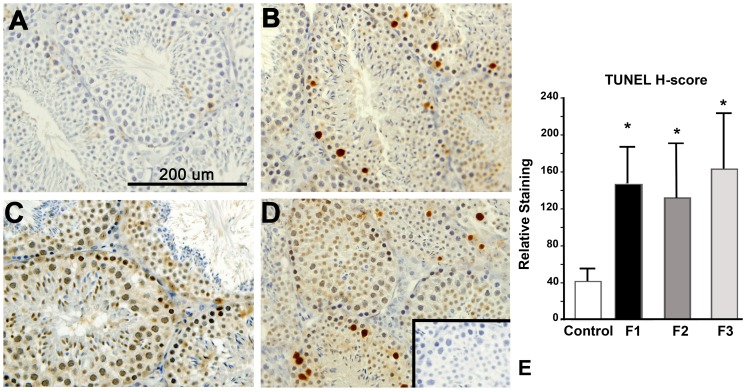
TUNEL staining in adult testis of control, F1, F2 and F3 males. TUNEL staining was minimal in control testis (A), but prominent in tissues from F1, F2 and F3 males (B–D). Semi-quantitative analysis of TUNEL staining is shown in E. Compared to control samples, *p*<0.01 for all toxicant exposed mice. Results are representative of at least 6 animals per group and from ≥3 more litters/group. Original magnification, 200x. **Inset**: Primary antibody omitted using the same tissue shown in Panel D.

Altered prostaglandin catabolism in mice with a history of toxicant exposure suggests that regulation of inflammatory processes within the testis is also disrupted in these animals. Since inflammatory conditions of the testis have previously been associated with apoptosis of germ cells leading to subfertility/infertility (reviewed by [42]), we conducted TUNEL staining and immunolocalization of cleaved caspase-3 in the testis of all animals. As shown in **Figure 7**, TUNEL staining was minimal in control testis, while staining was prominent in tissues from F1-F3 males. Caspase-3 immunohistochemistry revealed a similar pattern of staining (data not shown), demonstrating that the reduced sperm number in these animals correlates with increased apoptotic activity.

**Figure 8:**
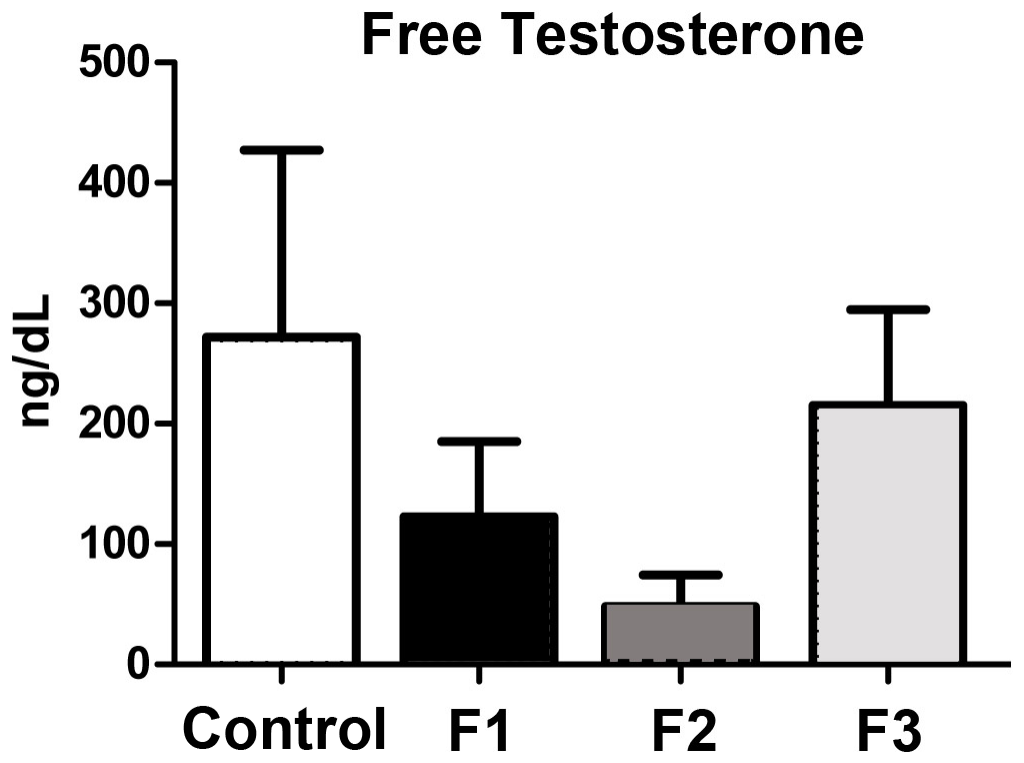
Radioimmunoassay of Free Testosterone. Free testosterone was measured in serum from control (N = 10), F1 (N = 14), F2 (N = 6) and F3 (N = 10) males. All samples were examined in duplicate and the results are presented as the mean±standard deviation. Levels of testosterone in experimental groups (F1-F3) was lower compared to control animals, but changes did not reach significance.

Since testicular inflammation has also previously been associated with impaired steroidogenesis in men [43], we next examined serum levels of testosterone in our animals. As shown in **Figure 8**, compared to control animals, circulating levels of testosterone were consistently lower in all mice with a direct (F1-F2) or indirect (F3) TCDD exposure, although the decrease did not reach statistical significance.

### Testicular Response to a Systemic Inflammatory Challenge

**Figure 9:**
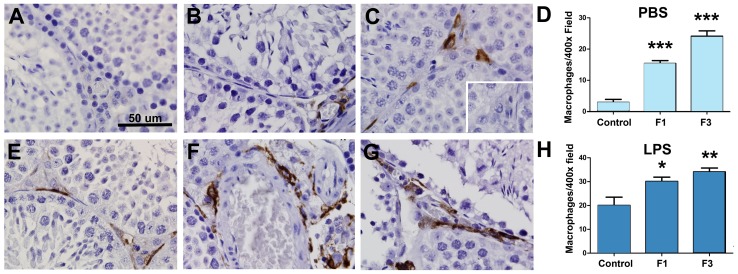
Assessment of macrophage number in adult murine testis of control, F1 and F3 males. The presence of testicular macrophages was assessed in the absence (A–D) and presence (E–H) of a low dose LPS challenge. Macrophages were rare in control males in the absence of LPS (Panel A), but were commonly found following the LPS challenge (Panel E). In contrast, macrophages were abundant in F1 males exposed only to PBS (Panel B) and were further increased by exposure to the LPS challenge (Panel F). Within the testis of F3 males, macrophages were abundant regardless of whether or not the mice had been challenged with LPS (Panels C and G). Semi-quantitative analysis of macrophage infiltrates is shown in Panels D and H. Results are representative of at least 6 animals per group and from ≥3 more litters/group. **Inset**: Primary antibody omitted using the same tissue shown in Panel C. Original magnification, 1000x. *p<0.05; **p<0.01 ***p<0.001.

In our previously published study of pregnant female mice with a history of developmental TCDD exposure (F1 females), we observed a doubling of the PTB rate in the presence of a systemic viral infection (parvovirus) and a 100% PTB rate in virus-free females following an inflammatory challenge with a single low-dose (200 ug/kg) peritoneal injection of LPS [18]. Control animals in this study never exhibited PTB, despite identical viral and bacterial challenges, suggesting that F1 females exhibit a heightened sensitivity to an inflammatory challenge. Significantly, this hyper-inflammatory phenotype was also present in F3 females, strongly suggesting germline epigenetic changes had occurred. Thus, in the current study, we examined whether alterations in sensitivity to infection-related inflammatory signals also affect the preconception testicular physiology of the F1 males. Equally important, we examined the same parameters in indirectly exposed F3 males in order to determine if a hyper-inflammatory testicular phenotype could be transmitted across multiple generations. Since altered macrophage numbers have specifically been shown to be associated with poor testicular health (reviewed by [44]), we used immunolocalization of F4/80 to examine the number of macrophages present in the testes of control, F1 and F3 males. As shown in **Figure 9A**, under non-stimulatory conditions, testicular immune cells are rare in control males and limited to the interstitial space containing the leydig cells. As expected, males with either a direct or indirect history of TCDD exposure exhibit enhanced numbers of resident macrophages compared to control animals (p<0.001); however, unexpectedly, F1 males, with a direct TCDD exposure, exhibited fewer resident macrophages compared to F3 males (with only an indirect TCDD exposure) (**Figure 9B–D**). Following an inflammatory challenge mediated by a single, low dose peritoneal injection of LPS, macrophages were present in all animals; however, semi-quantitative assessment revealed the numbers of macrophages present was significantly greater (p≤0.05) in mice with a TCDD exposure background compared to control testes (**Figure 9E–H**).

## Discussion

As a consequence of industrialization, human populations are exposed to an astonishing array of natural and synthetic chemicals, many of which have the biological potential to negatively impact our reproductive health. In addition to industrial sources of TCDD and dioxin-like compounds, cigarette smoke, automobile exhaust and even grilled meats can add other AhR-binding toxicants, including benzo(a)pryrene, to our daily exposome [45]. It is difficult to accurately determine the combinatorial reproductive effects of the numerous environmental agents we are exposed to at various stages of our lives; however, studies in experimental models clearly demonstrate the ability of various, individual endocrine disrupting toxicants to negatively affect fertility [18], [26], [46], [47]. Epidemiologic data of accidentally exposed human populations demonstrates that fertility in humans can be similarly, adversely affected [48], [49].

Relevant to the current study, infertility is common among humans, with an estimated incidence of 10% in reproductive age women [50] and a 12% occurrence among men [51]. Although multiple studies have indicated a decline in human sperm counts in recent decades (ie, [52], [53]), the heavy reliance of these studies on patients attending fertility clinics left open the possibility that the data was not representative of the general public [54]. However, a recent report from the European Union examining healthy young men has largely eliminated any doubt that sperm counts are declining in human populations [55]. Although the question remains regarding a potential relationship between declining sperm numbers and subsequent health of progeny in humans, several recent animal studies demonstrated that early life exposures to TCDD not only negatively affected the F1 offspring's individual fertility, but the health and reproductive capacity of future offspring as well [7], [18], [26].

The specific role(s) that toxicant exposure during development versus adulthood plays in either male or female reproductive health remains speculative. Nevertheless, a recent epidemiology study of women living within 4 kilometers of a municipal solid waste incinerator identified a significant increased risk for PTB [56], suggesting exposure to airborne toxicants adversely affected pregnancy outcomes. Presumably, the male partners of the women in this study also resided near the incinerator; however, the potential role of his exposure on pregnancy outcomes was not considered. Furthermore, while numerous epidemiologic studies support the relevance of a man's developmental exposure history to his adult sperm quality [57]–[59], these studies did not additionally assess the partner's pregnancy outcome. However, our murine studies revealed that early life TCDD exposure of male mice impacts inflammatory signaling at the maternal-fetal interface during late pregnancy, resulting in an elevated risk of spontaneous PTB [18], [20], [25]. To our knowledge, our studies were the first to identify a previous, developmental paternal TCDD exposure to a subsequent enhanced risk of PTB in his control mating partner. Further studies revealed inflammatory signaling within the largely paternally-derived placenta as a primary contributor to the paternal influence on PTB in our model [20], [25].

Since the contribution of the male to the placenta of pregnancy is delivered via the sperm, the current study was designed to examine whether adverse pregnancy outcomes in unexposed female mice could be traced to the preconception health of the testes in which the sperm developed. To this end, we utilized adult control males and adult males which were exposed to TCDD in utero on E15.5 (F1 males) in order to assess sperm number and morphology relative to the testicular environment and his partner's pregnancy outcome. Additionally, we examined two generations of the F1 male offspring in order to determine if either multi-generational or transgenerational effects occur following developmental TCDD exposure after sex determination. As shown in **Table 1**, we confirmed our previous studies that F1 male mice both exhibit infertility and, when fertile, present their unexposed female partners with a risk for PTB. Herein, we additionally examined the fertility and pregnancy outcomes of F2 and F3 males. It is interesting to note that F2 males exhibited the poorest reproductive health compared to other TCDD exposed groups. These data are consistent with our published observations in F2 females [18] and likely reflects an increased sensitivity of germ cells residing within the developing testes of the F1 fetus to direct TCDD exposure. Additionally, germ cells destined to become the F2 generation are also exposed to the secondary influence of the inflammatory testicular environment of the adult F1 males, which may further negatively impact the reproductive health of the adult F2 males.

Our current study additionally extends our previous observations by demonstrating a transgenerational, male germline-derived phenotype in the F3 generation, which included both sub-fertility and transmission of PTB risk (**Table 1**). Incremental improvements in fertility and gestation length were noted in each subsequent generation of mice examined, but these improvements did not reach significance. Reduced fertility and PTB risk likely persisted in F3 mice due to both the continued presence of significant morphological defects as well as biochemical abnormalities, such as the alteration in AhR expression identified herein (**Figure 4**). Our data is consistent with studies from others indicating heightened expression of spermatocyte AhR in association with infertility in men [10] and enhanced AhR expression in the spermatocytes of rats exposed in vivo to another environmental endocrine disruptor, BaP [60].

The testicular environment clearly impacts sperm development; therefore, in addition to examination of sperm, we also examined the expression of selected inflammatory biomarkers within the testis of control males and males with a direct or indirect toxicant exposure history. We found a near absence of PGDH expression in males with a history of toxicant exposure which correlated with a non-significant trend of increased PGE_2_ expression (**Figures 5** **and** **6**). These changes were associated with an increase in germ cell apoptosis (**Figure 7**), which was consistent with reduced sperm density in toxicant-exposed mice (**Figure 2**). Compared to unexposed mice, adult males with a direct (F1-F2) or indirect (F3) exposure also exhibited a non-significant reduction in serum levels of testosterone (**Figure 8**), a recognized consequence of chronic inflammation in men [43].

Finally, in our previous studies we demonstrated a transgenerational infertility phenotype in female mice of maternal lineage associated with a hyper-sensitivity to LPS-mediated PTB [18]. In the current study we found a similar transgenerational impact of development TCDD exposure in male mice, inherited through the paternal germ line. In addition to the paternal derived risk for PTB in control mating partners (**Table 1**), our current study also demonstrated that F1 and F3 male mice exhibit a hyper-responsiveness to an inflammatory challenge with LPS, most likely as a consequence of increased numbers of resident macrophages (**Figure 9**). While an acute challenge with LPS promoted the inflammatory testicular response regardless of the exposure history, F1 and F3 males exhibited elevated levels of resident macrophages and poor sperm morphology even in the absence of LPS challenge. Thus, while the “baseline” inflammatory and fertility profile in F1 and F3 animals was variable, *all* animals with a history of TCDD exposure exhibited a phenotype poised to exhibit an exaggerated inflammatory response following an exogenous challenge. These results are intriguing, as they raise the possibility that humans may also exhibit a varying ability to appropriately respond to common inflammatory/infectious agents which can have minimal or devastating effects depending on an individual's lifestyle and exposure history. Thus, individuals with a chronic hyper-inflammatory testicular phenotype would be at the greatest risk of reduced sperm quality and infertility. Furthermore, fertile individuals with an inflammatory testicular phenotype may be at greater risk of producing a hyper-inflammatory placental phenotype, thereby leading to adverse pregnancy outcomes in his partner.

Although the translation of our murine model to the human condition remains to be demonstrated, reducing male-derived PTB risk receives little or no attention during common fertility procedures such as intrauterine sperm injection (IUI) or intracytoplasmic sperm injection (ICSI). Nevertheless, the findings of our murine study clearly demonstrate that the testicular health of the male is fully capable of not only compromising his fertility but also the timing of birth in his female partner. Stated another way, our data suggest that the preconception health of the father may be indicative of the risk of PTB in his pregnant partner. If our studies hold true in men, intervention in the paternal partner designed to reduce preconception testicular inflammation may be a valuable tool to reduce the overall incidence of PTB in women. Equally important, our data suggests that optimizing the preconception health of the sperm may be particularly valuable as an adjunct therapy prior to ICSI or IUI.
